# CoNS blood culture isolates from very preterm infants: antibiotic susceptibility, synergy testing and *mecA* gene expression

**DOI:** 10.1093/jacamr/dlag058

**Published:** 2026-04-28

**Authors:** Zuzana Huncikova, Martin O Christensen, Runa Wolden, Hermoine J Venter, Karianne Wiger Gammelsrud, Jørgen V Bjørnholt, Elizabeth G Aarag Fredheim, Dag Harald Skutlaberg, Einar Nilsen, Kjersti Wik Larssen, Heidi J Espvik, Gunnar Skov Simonsen, Arild Rønnestad, Jorunn Pauline Cavanagh, Claus Klingenberg

**Affiliations:** Paediatric Department, Stavanger University Hospital, Stavanger, Norway; Department of Clinical Science, University of Bergen, Bergen, Norway; Research Group for Child and Adolescent Health, Department of Clinical Medicine, UiT-The Arctic University of Norway, Tromsø, Norway; Research Group for Child and Adolescent Health, Department of Clinical Medicine, UiT-The Arctic University of Norway, Tromsø, Norway; Research Group for Child and Adolescent Health, Department of Clinical Medicine, UiT-The Arctic University of Norway, Tromsø, Norway; Microbiology Department, Oslo University Hospital, Oslo, Norway; Medical Faculty, Institute for Clinical Medicine, University of Oslo, Oslo, Norway; Microbiology Department, Oslo University Hospital, Oslo, Norway; Medical Faculty, Institute for Clinical Medicine, University of Oslo, Oslo, Norway; MicroPop Research Group, Department of Pharmacy, Faculty of Health Sciences, UiT-The Arctic University of Norway, Tromsø, Norway; Department of Microbiology, Haukeland University Hospital, Bergen, Norway; Department of Microbiology, Møre and Romsdal Health Trust, Molde, Norway; Department of Medical Microbiology, St Olav University Hospital, Trondheim, Norway; Microbiology Department, Akershus University Hospital, Oslo, Norway; Department of Microbiology and Infection Control, University Hospital of North Norway, Tromsø, Norway; Research Group for Host-Microbe Interaction, Department of Medical Biology, Faculty of Health Sciences, UiT-The Arctic University of Norway, Tromsø, Norway; Medical Faculty, Institute for Clinical Medicine, University of Oslo, Oslo, Norway; Department of Neonatal Intensive Care, Clinic of Paediatric and Adolescent Medicine, Oslo University Hospital, Oslo, Norway; Research Group for Child and Adolescent Health, Department of Clinical Medicine, UiT-The Arctic University of Norway, Tromsø, Norway; Research Group for Child and Adolescent Health, Department of Clinical Medicine, UiT-The Arctic University of Norway, Tromsø, Norway; Department of Paediatric and Adolescent Medicine, University Hospital of North Norway, Tromsø, Norway

## Abstract

**Objectives:**

Previous reports suggest that neonatal sepsis with methicillin-resistant (MR) CoNS can be treated with first-generation cephalosporins. We have evaluated the *in vitro* effect of cefazolin and other antibiotics, synergy between β-lactams and aminoglycosides, and *mecA* expression in MR-CoNS isolates.

**Patients and methods:**

Antimicrobial susceptibility testing was performed in 167 CoNS isolates from 135 very preterm infants (<32 weeks gestation) with late-onset sepsis. Microbroth dilution chequerboard assay explored antibiotic synergy. PCR detected *mecA* and three aminoglycoside-modifying enzyme genes. Induction experiments evaluated if exposure to cefazolin induces resistance. RT-PCR determined *mecA* expression in selected MR-CoNS isolates with different cefazolin/oxacillin MICs.

**Results:**

All CoNS isolates were susceptible to amikacin and vancomycin. Most were phenotypically resistant to gentamicin (92%) and cefoxitin (93%), and 89% carried the *mecA* gene. MIC_90_ values of cefazolin and oxacillin were 16 mg/L and >256 mg/L, respectively. At the cefazolin breakpoint of 8 mg/L, 83% of isolates were susceptible *in vitro*. No antibiotic combinations showed synergy using the FIC index, but cefazolin combined with aminoglycosides had favourable susceptibility breakpoint index values. In a simplified synergy screen of ‘highly resistant’ isolates, growth was inhibited with a combination of gentamicin and cefazolin at sub-MIC concentrations in 8/42 (19%) isolates. Exposure to cefazolin sub-MICs caused reduced susceptibility in 1/30 (3%) tested isolates. The highest *mecA* expression was observed in isolates with high oxacillin/cefazolin MICs.

**Conclusions:**

CoNS isolates had lower MICs for cefazolin than oxacillin, and 83% were *in vitro* susceptible to cefazolin. A clinical trial comparing cefazolin with vancomycin as empirical therapy for suspected neonatal sepsis is warranted.

## Introduction

CoNS are the most prevalent pathogens causing late-onset sepsis (LOS) among very preterm infants (<32 weeks gestation) in high-income countries.^1–3^ In comparison with LOS caused by Gram-negative bacteria, CoNS-LOS attributable mortality is low and most CoNS-LOS episodes are mild to moderate. However, among the most vulnerable extremely preterm infants (<28 weeks gestation), CoNS may cause sepsis with a fatal outcome. Empirical regimens with adequate coverage for both low-virulent CoNS and high-virulent Gram-negative pathogens are therefore important.

The majority (>80%) of CoNS blood culture isolates from neonatal intensive care unit (NICU) patients are *in vitro* methicillin-resistant (MR), but most are susceptible to vancomycin.^4–6^ There are variations in vancomycin use after the first week of life.^7–9^ Empirical vancomycin therapy for preterm infants with suspected LOS is controversial due to the relatively low virulence of CoNS, the risk of vancomycin toxicity, additional blood sampling for therapeutic drug monitoring, and because vancomycin is less rapidly bactericidal than β-lactams in the treatment of methicillin-susceptible staphylococci.^9–13^ Indiscriminate use of vancomycin is also linked to the emergence of vancomycin-resistant organisms.^14,15^ Vancomycin, particularly when combined with aminoglycosides, has a higher nephrotoxic and ototoxic potential in preterm infants compared with β-lactams.^16–18^

Several large multicentre studies in preterm infants have failed to demonstrate a survival benefit with empirical vancomycin therapy if MR-CoNS are detected in the blood culture, compared with empirical β-lactam antibiotics combined with an aminoglycoside or delayed vancomycin therapy.^11,19^ In Norway, we found a low CoNS-LOS attributable mortality across all regions, despite marked variations in vancomycin use.^7^ Some studies report that MR-CoNS sepsis cases can still be successfully treated with first-generation cephalosporins, also providing additional Gram-negative coverage in comparison with vancomycin.^20–22^

In this study we investigated antibiotic susceptibility patterns and the presence of genes encoding antibiotic resistance (ARGs) to β-lactams and aminoglycosides in a large cohort of CoNS isolates obtained from blood cultures of very preterm infants with LOS. We aimed specifically to evaluate the *in vitro* effect of cefazolin, potential synergies between β-lactam antibiotics and aminoglycosides, potential cefazolin induction of resistance in CoNS as well as the relationship between *mecA* gene expression and phenotypic susceptibility.

## Methods

### Setting, data source and ethics

Very preterm infants with CoNS-LOS were identified and clinical data obtained from the Norwegian Neonatal Network (NNN). NNN is a national population-based register collecting person-identifiable data from all 20 Norwegian NICUs without need for consent, according to regulations for the Medical Birth Registry of Norway and the Norwegian Personal Health Data Filing System Act.^23^ The Regional Ethical Committee for Medical and Health Research Ethics approved this study (REK Helse Sør-Øst 2012/944-1).

### Population, data and definitions

In a previous NNN-register study from 2009 to 2018, we identified all very preterm infants with LOS. Among 5232 NICU-admitted very preterm infants, 224 (4.3%) had one or more CoNS-LOS episodes.^1^ Growth of CoNS in blood culture was classified as LOS when the infant had clinical symptoms after 3 days of life, received a minimum of 5 days of antibiotic therapy and had an elevated C-reactive protein (CRP >10 mg/L).^1^ In the current study we contacted all relevant microbiological laboratories and identified 167 CoNS isolates in 135 patients from the original cohort. All isolates were shipped to the laboratory at UiT (The Arctic University of Norway), and re-speciated using MALDI-TOF MS (MALDI Biotyper, Compass IVD software v4.2) with a score ≥2.0 accepted as species identification.

### Antimicrobial susceptibility testing

Antimicrobial susceptibility testing (AST) was performed for 11 different antibiotics. Disc diffusion was performed according to the EUCAST methods for clindamycin, gentamicin, amikacin, linezolid, ciprofloxacin, cefoxitin and rifampicin.^24,25^ Cefoxitin disc diffusion reliably predicts MR. MIC values of cefazolin and oxacillin were determined using agar gradient tests according to the manufacturer’s instructions (Liofilchem, Italy). For vancomycin, the broth microdilution (0.016–256 μg/mL) assay was performed (Liofilchem, Italy). Except for cefazolin and oxacillin, all susceptibility results were interpreted according to EUCAST clinical breakpoints v15.0.^25^ The breakpoint for cefazolin was set to 8 mg/L, as suggested by others.^21,26^ We used control strains (ATCC 25922 *Escherichia coli* and ATCC 29213 *Staphylococcus aureus*) for both AST and synergy testing.

### Antibiotic synergy testing

Broth microdilution chequerboard assays were used to explore possible synergistic effects between cefazolin-amikacin, cefazolin-gentamicin, oxacillin-amikacin and oxacillin-gentamicin, as described previously.^27^ Briefly, testing was done in CAMHB and standard 2-fold dilutions for both drugs. Concentration ranges were adjusted to the MICs for each isolate. Each plate included testing of each drug alone to confirm its MIC, and positive and negative controls (inoculum only and drug only, respectively). Plates were seeded with a bacterial inoculum of approximately 5 × 10^5 ^cfu/mL. Plates were incubated at 37°C under humidified conditions for 20 h and visually examined. Each assay was repeated with at least three biological replicates. FICs were calculated for each drug concentration combination that inhibited growth to determine the FIC index (FICI). The FICI was interpreted as synergy (mean FICI ≤0.5), no interaction (mean FICI >0.5 to ≤4.0) or antagonism (mean FICI >4.0).^28^ The mean susceptibility breakpoint index (SBPI) was calculated and interpreted as poor (<2), good (2–50) or very good (50–100).^28^ All four antibiotic combinations were tested on four selected isolates (two *Staphylococcus epidermidis* and two *Staphylococcus capitis*). Moreover, two isolates were tested in a full chequerboard assay with the combination cefazolin/gentamicin only.

We then performed a simplified synergy test using the same broth microdilution assay on 42 CoNS isolates to further explore possible synergies with cefazolin/gentamicin. The isolates were selected based on the following criteria: (i) gentamicin MIC ≥ 32 mg/L; (ii) cefazolin MIC between 4 and >256 mg/L; and (iii) *mecA* positive. A fixed gentamicin concentration of 12 mg/L was used in all synergy experiments, approximating high peak (*C*_max_) serum levels in infants.^29^ Cefazolin concentrations were one and two dilutions below MIC up to MIC 8 mg/L. For isolates with cefazolin MIC > 8 mg/L, we examined synergy using cefazolin 4 and 8 mg/L. We did not use higher cefazolin concentrations since neonatal dosing models have reported it is possible to achieve cefazolin concentrations above 8 mg/L for 60% of the time (T/MIC), but not for concentrations above 16 mg/L.^30^ This simplified synergy testing was performed with three biological replicates.

### Induction experiments

Cefazolin can induce bacterial resistance in Gram-negative bacteria by enhanced *AmpC* expression.^31^ We evaluated whether a similar phenomenon occurs in CoNS. We selected 30 *mecA*-positive CoNS isolates (15 *S. epidermidis* and 15 *S. capitis*) with cefazolin MIC 4 mg/L, based on the agar gradient test results (Resistant = MIC > 8 mg/L). All isolates were grown overnight in CAMHB (‘control’) or CAMHB supplemented with a cefazolin sub-MIC of 1 mg/L (‘sub-MIC’). The cefazolin MIC for each overnight culture was analysed using the micro broth dilution assay, with three biological replicates. A two-dilution increase in cefazolin MIC from ‘control’ to ‘sub-MIC’ in a minimum two out of three replicates was defined as evidence of induction.

### Antibiotic resistance genes

PCR was used to determine the presence of the methicillin resistance gene *mecA* as well as three genes encoding aminoglycoside-modifying enzymes (AMEs): *aac(6′)-Ie-aph(2'′)-Ia*, *ant(4′)-Ia* and *aph(3′)-IIIa*. In brief, bacterial DNA was isolated by boiling bacterial colonies resuspended in TE buffer (10 mM Tris-HCl, 1 mM EDTA, pH 8.0; Sigma-Aldrich, USA) for 10 min.^32^ Genomic DNA was verified by a PCR targeting the universal 16S rRNA gene using OneTaq Master Mix (New England Biolabs, USA). Primer combinations and PCR conditions for the detection of ARGs are listed in Table S1 (available as Supplementary data at *JAC-AMR* Online).

### *mecA* expression

*mecA* expression in nine *S. epidermidis* and/or *S. capitis* isolates, with low, medium and high oxacillin/cefazolin MIC values, was investigated by RT-PCR. Four MRSA isolates, obtained from the national MRSA reference centre, were included for comparison. Bacteria were grown in CAMHB to an optical density of 0.5–0.8 at OD_600_ (Pharmacia Biotech, Sweden). Samples were harvested and stabilized using RNA protect (QIAGEN, Germany), and total RNA was isolated using the RNeasy Mini Kit (QIAGEN, Germany), according to the manufacturer’s instructions. Genomic DNA was eliminated using the Heat&Run gDNA Removal Kit (ArcticZymes, Norway). cDNA was synthesized using the High-Capacity cDNA Reverse Transcription Kit (Thermo Fisher Scientific, USA). Synthesized cDNA was used as a template for quantification of *mecA* by quantitative PCR (qPCR) using the Takyon Low ROX SYBR 2X MasterMix Blue dTTP (Eurogentec, Belgium) and the LightCycler^®^ 96 Instrument (Roche Diagnostics GmbH, Germany) with Instrument Software Version 1.02.00.0086. Data analysis was conducted using the LightCycler^®^ 96 Application Software Version 1.1.0.1312. Quantification of *mecA* expression was performed relative to the housekeeping gene gyrase subunit B (*gyrB*). The ΔCt values were calculated as the difference between the Ct values of the target gene (*mecA*) and the reference gene (*gyrB*). The relative expression levels were then calculated using the formula: fold change = 2^−ΔCt^

### Statistical methods

Statistical analyses were performed with SPSS 29.0 (SPSS, USA). Results are presented as medians (IQRs) or proportions (%). Differences between groups were analysed with non-parametric tests for continuous variables, and Fisher exact or chi-square tests for categorical data, as appropriate. Two-tailed *P* values <0.05 were considered statistically significant.

## Results

### Demographics

Demographic data are presented in Table 1. Most infants were born before 28 weeks of gestation. There was a high rate of comorbidities including 55/135 (41%) infants with severe bronchopulmonary dysplasia, and 16/135 (12%) infants who were treated for retinopathy of prematurity. Median (IQR) duration of hospitalization for all infants was 99 (62–130) days. All infants received an aminoglycoside (gentamicin or tobramycin) at some point. The types of β-lactams administered varied considerably. A total of 111/135 (82%) infants were administered vancomycin, with median (IQR) treatment duration of 8 (5–12) days.

**Table 1.** Demographic data for 135 very preterm infants with CoNS late-onset sepsis

	*S. epidermidis* *n* = 61 infants	*S. capitis* *n* = 55 infants	Other CoNS *n* = 19 infants
Birth weight, g	790 (672–1063)	728 (617–920)	775 (637–1106)
Gestational age, wk	26 + 5 (25 + 1 to 28 + 5)	26 + 0 (24 + 4 to 27 + 3)	26 + 0 (24 + 4 to 28 + 4)
Small for gestational age	17/61 (28)	20/55 (36)	4/19 (22)
C-reactive protein at first LOS episode, mg/L	56 (36–77)	45 (36–103)	59 (35–82)
Total duration of antibiotics after first week of life	8 (4–12)	8 (4–12)	7 (4–9)
Total duration of indwelling central lines	5 (0–25)	5 (0–17)	6 (0–11)
Received vancomycin	43/61 (70)	43/55 (78)	14/19 (74)
Death attributable to CoNS LOS	4/61 (7)	1/55 (2)	1/19 (5)
Death before discharge	7/61 (11)	3/55 (5)	3/19 (16)

All data are median (IQR) or number (%).

### Species determination and antimicrobial susceptibility

**Figure 1.**
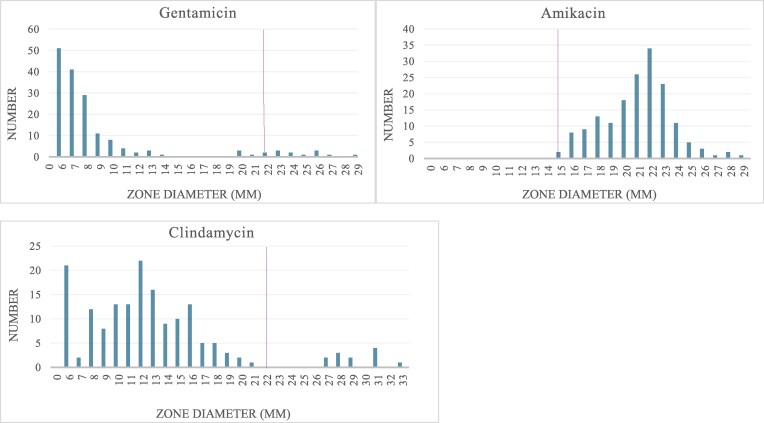
Distribution of inhibition zones for gentamicin, amikacin and clindamycin in the study collection. Purple vertical lines mark the clinical breakpoints.

**Figure 2.**
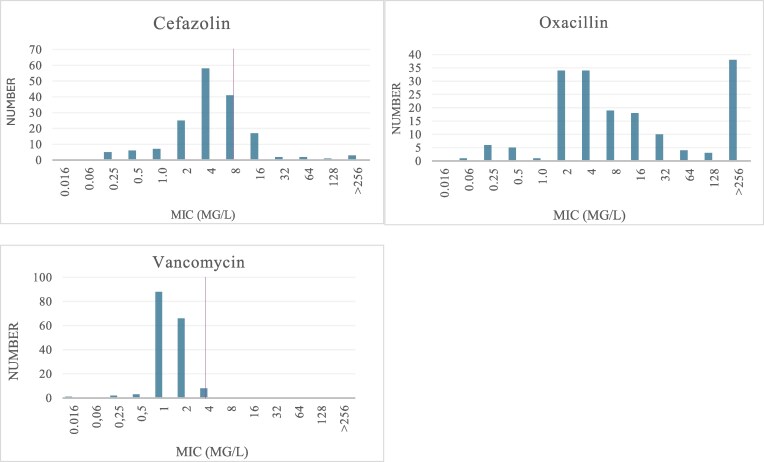
Agar gradient MIC distribution for cefazolin, oxacillin and vancomycin in the study collection. Purple verticallines mark the clinical breakpoint for vancomycin and the suggested clinical breakpoint for cefazolin. Oxacillin epidemiological cut-off values are 0.5 mg/L and 1 mg/L for the most prevalent CoNS species, *S. epidermidis* and *S. capitis*, respectively.

The two most prevalent species were *S. epidermidis* (71/167; 43%) and *S. capitis* (70/167; 42%). The remaining CoNS isolates were *Staphylococcus warneri* (20/167; 12%), *Staphylococcus hominis* (4/167; 2%) and *Staphylococcus haemolyticus* (2/167, 1%). Susceptibility patterns are shown in Table 2. All isolates were sensitive to vancomycin and amikacin. According to EUCAST clinical breakpoints, most isolates were phenotypically resistant to gentamicin (92%) and cefoxitin (93%). There was some divergence in susceptibility patterns between *S. epidermidis* and *S. capitis.* Rates of resistance to rifampicin (6% versus 0%) and ciprofloxacin (56% versus 1.4%) were higher in *S. epidermidis* than in *S. capitis* isolates. At cefazolin breakpoint 8 mg/L,^26^ 139/167 (83%) of CoNS isolates were susceptible. Cefazolin susceptibility rates were higher among *S. epidermidis* isolates (66/71; 93%) than among CoNS non-epidermidis isolates (73/96; 76%) (*P* < 0.001). The MIC_50_ and MIC_90_ of oxacillin were 8 mg/L and >256 mg/L, respectively. In comparison, the MIC_50_ and MIC_90_ of cefazolin were 4 mg/L and 16 mg/L, respectively. The distributions of inhibition zone diameters and MICs are shown in Figures 1 and 2.

**Table 2.** Phenotypic and genotypic antimicrobial susceptibility profile of 167 CoNS blood culture isolates obtained from 135 very preterm infants

	All CoNS *n* = 167	*S. epidermidis* *n* = 71	*S. capitis* *n* = 70	*S. warneri* *n* = 20	*S. hominis* *n* = 4	*S. haemolyticus* *n* = 2
Methicillin resistance gene						
• *mecA*	149/167 (89)	60/71 (85)	67/70 (96)	17/20 (85)	3/4 (75)	2/2 (100)
Cefazolin-R (MIC > 8 mg/L)	28/167 (17)	5/71 (7)	12/70 (17)	9/20 (45)	1/4 (25)	1/2 (50)
Cefoxitin-R (ZD < 22 mm/<27 mm *S. epidermidis*)	155/167 (93)	63/71 (89)	70/70 (100)	17/20 (85)	3/4 (75)	2/2 (100)
Aminoglycoside-modifying enzyme genes						
• *aac(6′)-Ie-aph(2′)-Ia*	145/167 (87)	64/71 (90)	58/70 (83)	18//20 (90)	3/4 (75)	2/2 (100)
• *ant(4′)-Ia*	28/167 (17)	22/71 (31)	4/70 (6)	2/20 (10)	1/4 (25)	0/2 (0)
• *aph(3′)-IIIa*	1/167 (1)	0/71 (0)	0/70 (0)	0/20 (0)	0/4 (0)	1/2 (50)
Gentamicin-R (ZD < 22 mm)	154/167 (92)	63/71 (89)	69/70 (99)	18/20 (90)	2/4 (50)	2/2 (100)
Amikacin-R (ZD < 15 mm)	0/167 (0)	0/71 (0)	0/70 (0)	0/20 (0)	0/4 (0)	0/2 (0)
Vancomycin-R (MIC > 4 mg/L)	0/167 (0)	0/71 (0)	0/70 (0)	0/20 (0)	0/4 (0)	0/2 (0)
Clindamycin-R (ZD < 22 mm)	28/167 (17)	22/71 (31)	3/70 (4)	1/20 (5)	2/4 (50)	0/2 (0)
Rifampicin-R (ZD < 30 mm)	7/167 (4)	4/71 (6)	0/70 (0)	2/20 (10)	1/4 (25)	0/2 (0)
Ciprofloxacin-R (ZD < 22 mm)	44/167 (26)	40/71 (56)	1/70 (1)	0/20 (0)	2/4 (50)	1/2 (50)
Linezolid-R (ZD < 20 mm)	4/167 (2)	2/71 (3)	0/70 (0)	2/20 (10)	0/4 (0)	0/2 (0)

R, resistant; ZD, zone diameter.

### Synergy testing

Synergy testing was performed on *S. epidermidis* and *S. capitis* isolates with low (4−8 mg/L) or high (64–128 mg/L) oxacillin MICs (Table 3). With a conservative FICI interpretation, none of the four antibiotic combinations showed synergy. However, for the combination of cefazolin and gentamicin/amikacin, the lower boundary of the range of the FICI was around 0.5 (0.3–1) for all four strains. These combinations also gave favourable SBPI values of between 3.4 and 42.7.

**Table 3.** Synergy testing with chequerboard assay of six selected isolates for combinations of β-lactam and aminoglycoside antibiotics

Species (strain ID)	Drug combination (A/B)	MIC drug A, mg/L Median (range)	MIC drug B, mg/L Median (range)	FICI^a^ Mean (range)	SBPI^b^ Mean (range)^a^
*S. epidermidis* (117)	OXA/AMK	128 (64–128)	16 (16–32)	1.76 (0.56–8)	1.03 (1.03)
	OXA/GEN	64 (64)	64 (64–128)	2.5 (0.6–8)	0.1875 (0.125–0.3125)
	CFZ/AMK	16 (16)	32 (32)	0.73 (0.51–1)	**33 (33)**
	CFZ/GEN	16 (16)	128 (64–128)	0.76 (0.5–1.25)	**2.72 (2.06–4.06)**
*S. epidermidis* (13)	OXA/AMK	4 (4)	1 (1–2)	2.69 (0.63–8.25)	**17 (17)**
	OXA/GEN	4 (4)	8 (8)	2.51 (0.63–8.1)	1.5 (1.5)
	CFZ/AMK	2 (2–4)	2 (2–4)	2.21 (0.5–8.13)	**40 (32–48)**
	CFZ/GEN	2 (2)	8 (8)	2.61 (0.63–8.1)	**8.0 (8.0)**
*S. capitis* (12)	OXA/AMK	8 (4–8)	2 (2)	1.27 (0.63–4.13)	**10 (10)**
	OXA/GEN	8 (4–8)	16 (16)	1.27 (0.63–4)	**2.13 (2.13)**
	CFZ/AMK	4 (4)	2 (2–4)	1.54 (0.31–4.1)	**42.67 (40–48)**
	CFZ/GEN	4 (4)	16 (16)	1.8 (1–4)	**32.13 (32.13)**
*S. capitis* (130)	OXA-AMK	64 (64)	2 (2)	2.46 (0.5–8.13)	**16.06 (16.06)**
	OXA/GEN	64 (64)	16 (8–16)	2.45 (0.25–8.1)	0.81 (0.31–1.06)
	CFZ/AMK	8 (8)	2 (2–4)	1.14 (0.5–2.25)	**38.7 (36–40)**
	CFZ/GEN	8 (8)	16 (16–32)	1.07 (0.53–2.03)	**32.13 (32.13)**
*S. capitis* (30)	CFZ/GEN	4	32	1.28	**16 (16)**
*S. epidermidis* (51)	CFZ/GEN	4	32	1.16	**2.25 (2.25)**

AMK, amikacin; CFZ, cefazolin; GEN, gentamicin; OXA oxacillin.

^a^Synergy was defined as a FICI  ≤0.5, indifference as a FICI between >0.5 and 4, and antagonism as a FICI >4.

^b^Susceptibility breakpoint index (SBPI) was calculated from the lowest concentration of the drug combinations that gave inhibition in each replicate and averaged:

(breakpoint drug A/MIC drug A in combination) + (breakpoint drug B/MIC drug B in combination). SBPI <2 = poor; 2–50 = good; 50–100 = very good.

For calculation of SBPI, we used an epidemiological cut-off value for OXA (>0.5 mg/L), EUCAST clinical breakpoints for AMK (>16 mg/L) and GEN (>2 mg/L), and a suggested clinical breakpoint for CFZ (>8 mg/L). Combinations with SBPI in the category ‘good’ are marked in bold.

In the simplified synergy screen, we included 42 isolates with a ‘resistant’ profile (Table S2). All had gentamicin MICs ≥32 mg/L. Cefazolin MICs were 4 mg/L (*n* = 13), 8 mg/L (*n* = 21) and ≥16 mg/L (*n* = 8). Using the fixed gentamicin concentration (12 mg/L) combined with variable sub-MIC cefazolin concentrations, growth was inhibited in 3/8 (38%) isolates with a cefazolin MIC ≥ 16 mg/L. Among the isolates with a cefazolin MIC ≤ 8 mg/L, growth was inhibited in 5/34 (15%) isolates.

### Correlation between phenotypic and genotypic findings

The *mecA* gene was identified in 149/167 (89%) CoNS isolates. There was good concordance between the cefoxitin disc diffusion test and *mecA* results. In *S. epidermidis*, 63/71 isolates were MR according to the cefoxitin disc diffusion, and 60/71 carried the *mecA* gene. In *S. capitis*, 70/70 isolates were MR according to the cefoxitin disc diffusion, and 67/70 carried the *mecA* gene.

The *aac(6′)-Ie-aph(2′)-Ia* gene was identified in 145/167 (87%), *ant(4′)-Ia* in 28/167 (17%) and *aph(3′)-IIIa* in 1/167 (0.6%) CoNS isolates. Overall, 140/154 (91%) of isolates phenotypically identified as gentamicin resistant carried the *aac(6′)-Ie-aph(2′)-Ia* gene, and 18% (25/140) also carried the *ant(4′)-Ia* gene. In contrast, all CoNS isolates containing AME-encoding genes were classified as amikacin susceptible.

### Induction experiments

Among 30 *mecA*-positive CoNS isolates, only 1 isolate showed evidence of possible cefazolin induction with a MIC at 4 mg/L when grown without antibiotic exposure and a MIC of 32 mg/L after sub-MIC cefazolin exposure (Table S3).

### mecA expression

**Figure 3.**
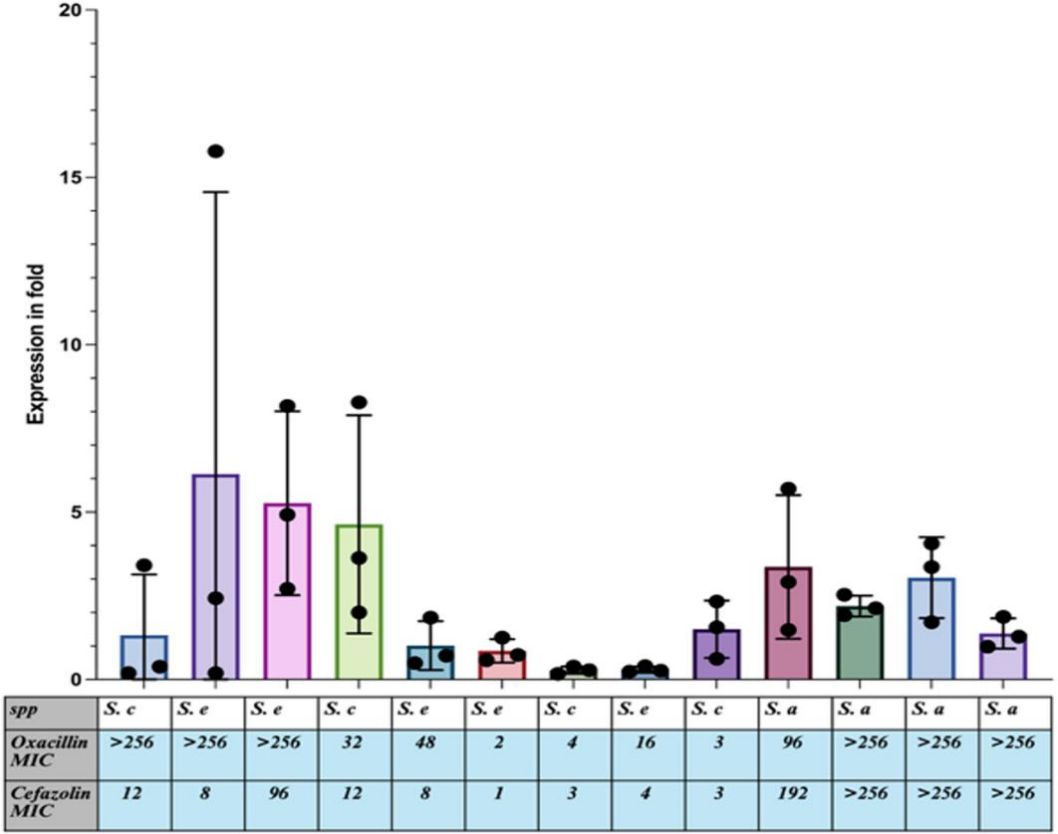
Fold changes in *mecA* gene expression relative to the housekeeping gene gyrase subunit B (*gyrB*) (*y*-axis) in relation to oxacillin MIC values in selected oxacillin-resistant CoNS and MRSA isolates. *S. c*, *Staphylococcus capitis*; *S. e*, *Staphylococcus epidermidis*; *S. a*, *Staphylococcus aureus*.

Results from the *mecA* expression analyses for nine CoNS isolates with high, medium and low oxacillin/cefazolin MIC values, and the four MRSA isolates, are presented in Figure 3 and Table S4. Compared with the housekeeping gene *gyrB*, the three highest *mecA* expression values (>4-fold increase) were observed in CoNS isolates with oxacillin and cefazolin MICs ranging from 32 to 256 mg/L and 8 to 96 mg/L, respectively. The three lowest *mecA* expression values (<1-fold increase) were observed in CoNS isolates with oxacillin and cefazolin MICs ranging from 2 to 16 mg/L and 1 to 4 mg/L, respectively. The four MRSA isolates showed *mecA* expression levels that were 1.4- to 3.4-fold higher compared with the CoNS isolates.

## Discussion

In this study, we report detailed data on antibiotic susceptibility, potential synergy between β-lactams and aminoglycosides, and *mecA* expression in CoNS blood culture isolates from a large cohort of very preterm infants with LOS. To our knowledge, no previous studies have investigated the potential synergy of common LOS empirical antibiotic combinations in this vulnerable population, where CoNS is the major causative pathogen.^1,7,33^ We found that around 80% of MR-CoNS isolates were phenotypically susceptible to cefazolin. Higher *mecA* expression values were associated with higher MIC values for both oxacillin and cefazolin, analysed in a selected number of MR-CoNS isolates. With a conservative FICI interpretation, none of the four antibiotic combinations tested showed synergy. However, when evaluating the lower boundary of FICI values, the SBPI results and our simplified synergy screen in a group of ‘more resistant’ isolates we identified a potential synergy between cefazolin and gentamicin/amikacin. The restricted number of isolates evaluated requires caution when interpreting these results.

In line with several other reports, CoNS isolates from very preterm infants in our study were often MDR.^6^ Indeed, the vast majority of isolates were *in vitro* both MR and resistant to many other antibiotics. However, susceptibility testing revealed much lower MIC values for cefazolin than oxacillin. No studies have directly compared PBP-2a affinity between cloxacillin and cefazolin, but it has been suggested that some of the older β-lactams, such as cefazolin, have a higher binding affinity for PBP-2a.^21^ Neonatal dosing models have reported cefazolin concentrations above 8 mg/L for 60% of the time.^30^ Based on these data, and *in vivo* evidence suggesting that cefazolin has clinically relevant activity against *mecA*-positive CoNS isolates, a cefazolin breakpoint of 8 mg/L has been suggested for CoNS.^26,30^ In contrast, EUCAST and CSLI recommend that susceptibility of staphylococci to cephalosporins is inferred from cefoxitin susceptibility or the presence of *mecA*.^34^ Studies on cefazolin in neonatal CoNS infections are not included in the evidence base for EUCAST/CSLI breakpoints for staphylococci. The recommended EUCAST/CLSI cefazolin breakpoints for other bacteria include comments on conventional and high adult cefazolin doses (2 g every 8 h). Normal cefazolin dosing for infants (25–50 mg/kg) equals the high adult cefazolin dose.^26^ Our induction experiments also suggest that cefazolin sub-MIC rarely causes induction of resistance in *mecA*-positive CoNS.

The majority of CoNS isolates were gentamicin-resistant, as described in other studies.^35,36^ In contrast, all CoNS isolates were susceptible to amikacin, despite 87% harbouring *aac(6′)-Ie-aph(2′)-Ia*, which encodes the bifunctional AME.^37–39^ Discordance between phenotypic susceptibility and detection of AME-encoding genes is known.^40^ The AME subtype AAC(6*′*)-Ib, acetylating amikacin in the 6*′*-*N* position, is the major AME causing amikacin resistance in Gram-negative pathogens.^41^ However, the bifunctional AME, more commonly seen in Gram-positive pathogens, contains the AME subtype AAC(6*′*)-Ie. Our data suggest that amikacin is more resistant to the inactivating effect of the bifunctional AME than gentamicin.^37,42^ Amikacin is currently used for combination therapy of neonatal sepsis in several NICUs.^43,44^ However, a previously observed increase in amikacin resistance among CoNS associated with increased amikacin use is of concern.^35,45^

More than three-quarters of the infants with CoNS-LOS had been treated with vancomycin and all included CoNS isolates were vancomycin-susceptible, as reported in other studies.^4,35^ The WHO categorizes antimicrobials into ‘Access’, ‘Watch’ and ‘Reserve’ categories with the recommendation to limit the use of ‘Watch’ and ‘Reserve’ agents where possible.^46^ Vancomycin is classified in the ‘Watch’ category, and ‘Access’ type antibiotics like cefazolin should be preferred, if possible. CoNS seldom cause severe sepsis and there is time to switch to vancomycin if the patient is not responding to empirical therapy and the identified bacterium is resistant. This approach could reduce potential ototoxic and nephrotoxic effects associated with vancomycin, which are further aggravated if vancomycin is administered together with an aminoglycoside.^11,16,17,19,47,48^ Moreover, this approach can also decrease overuse of vancomycin, a main risk factor for the emergence of VRE and staphylococcal strains with reduced vancomycin susceptibility.^14,15,49^

The intention behind combining two antibiotics for empirical LOS therapy in preterm infants is mainly to cover for Gram-negative (often with an aminoglycoside) and Gram-positive (often with a β-lactam or a glycopeptide) pathogens.^50–52^ Standard AST does not consider whether the combination of two antibiotics may have additive or synergistic activity sufficient to be effective against pathogens resistant to each antibiotic individually.^53^

*In vitro* synergistic effects have been reported for β-lactam + aminoglycoside combinations against Gram-negative^29,54^ and Gram-positive bacteria.^55,56^ A proposed mechanism is increased aminoglycoside uptake when combined with cell wall disruptive β-lactams.^55^ In neonatal sepsis pathogens, *in vitro* synergy of antibiotic combinations has hitherto been shown for ampicillin and gentamicin against Group B streptococci and Gram-negatives.^53,57,58^ Moreover, one study from 2013 reported *in vitro* synergy between oxacillin and gentamicin against oxacillin-resistant CoNS.^59^ We tested different combinations of β-lactams with aminoglycosides for synergistic effects, reflecting common empirical therapies.^60^ Using a conservative FICI interpretation, no synergy (FICI < 0.5) was observed. Some suggest the SBPI is a better tool to categorize drug combination synergy.^61^ Considering SBPI, the majority of our antibiotic combinations are placed in the ‘good’ category. In the simplified synergy testing, 3/8 highly resistant CoNS isolates were inhibited with sub-MIC cefazolin concentrations when combined with gentamicin at 12 mg/L.

There is a paucity of clinical data on the response to cefazolin therapy in preterm infants with MR-CoNS. However, two Dutch studies have demonstrated safety and efficacy of first-generation cephalosporin (cefalotin and cefazolin) treatment as first-line empirical therapy for MR-CoNS-LOS.^20,22^ The overall safety of anti-staphylococcal penicillins rather than vancomycin to provide empirical Gram-positive coverage during LOS evaluation in centres with low MRSA prevalence has been demonstrated, without evidence of increased duration of bacteraemia, infectious complications or mortality.^14,62,63^

In contrast to MRSA, *mecA* presence in CoNS does not necessarily result in β-lactam resistance.^64–68^ In our study, cefoxitin resistance correlated well with *mecA* gene presence, but this was not the case for cefazolin. In line with our study, a recent study from Australia found that 75% of CoNS bacteraemia isolates had a cefazolin MIC ≤ 8 mg/L, without correlation to the presence of the *mecA* gene.^26^ However, our *mecA* expression studies indicated that isolates with high cefazolin MIC values, although rare, also displayed higher *mecA* expression values.

The strength of this study is the inclusion of a large number of blood culture CoNS isolates from NICUs across Norway with clear criteria defining them as sepsis associated. All isolates underwent extensive AST and detection of relevant ARGs. Our study also has limitations. First, although it includes clinical data, the study is based on *in vitro* experimental analyses. Second, time–kill assays, the preferred method for synergy testing, is very labour intensive. We chose to use broth microdilution assays to look for trends in synergistic effects. Finally, we did not perform WGS. Thus, we could not assess all potentially clinically relevant ARGs and cannot rule out that some isolates were clonally related. However, isolates came from eight different NICUs and we specifically selected isolates with a broad range of oxacillin and cefazolin MIC values for synergy testing.

### Conclusions

In our study, 83% of the isolates had a cefazolin MIC ≤ 8 mg/L. We found lower *mecA* gene expression in CoNS isolates with low cefazolin/oxacillin MICs. Our data indicate that cefazolin is active *in vitro* against a majority of CoNS isolates. Previous clinical studies support that empirical cefazolin therapy may reduce vancomycin use, an important antimicrobial stewardship target in NICUs. A large randomized clinical trial of cefazolin versus vancomycin, both combined with an aminoglycoside, for empirical LOS therapy in the NICU should be considered to evaluate clinical efficacy and drug-associated toxicity.

## Supplementary Material

dlag058_Supplementary_Data

## Supplementary data

Tables S1 to S4 are available as Supplementary data at *JAC-AMR* Online.
