# Supplementary material for: CoNS blood culture isolates from very preterm infants: antibiotic susceptibility, synergy testing and mecA gene expression
Source: JAC Antimicrob Resist. 2026 Apr 28;8(2):dlag058. doi: 10.1093/jacamr/dlag058 (PMC13121896; doi:10.1093/jacamr/dlag058)
Supplement: dlag058_Supplementary_Data [file dlag058_supplementary_data.docx]

**Supplementary Table 1**. Overview of primer sequences and annealing temperature.

| **Gene** | **Sequence (5’-3’)** | **Annealing temperatures** |
| --- | --- | --- |
| *MecA FW^1^* | AAAATCGATGGTAAAGGTTGGC |  |
| *MecA RV^1^* | AGTTCTGCAGTACCGGATTTGC | 58°C |
| *aac(6) FW^2^* | CAGAGCCTTGGGAAGATGAAG |  |
| *aac(6) RV^2^* | CCTCGTGTAATTCATGTTCTGGC | 58°C |
| *ant(4) FW^2^* | CAAACTGCTAAATCGGTAGAAGCC |  |
| *ant(4) RV^2^* | GGAAAGTTGACCAGACATTACGAACT | 59°C |
| *aph(3) FW^2^* | GGCTAAAATGAGAATATCACCGG |  |
| *aph(3) RV^2^* | CTTTAAAAAATCATACAGCTCGCG | 56°C |
| *RT-MecA-FW^3^* | TGGGTACAAGATGATACCTT |  |
| *RT-MecA-RV^3^* | AATAGATGTGAAGTCGCTTT | 60°C |
| *16S rRNA FW* | AGA GTT TGA TCC TGG CTC AG | 58°C |
| *16S rRNA RV* | ACG GCT ACC TTG TTA CGA CTT |  |
| *RT-gyrB FW s.a.* | AGGTCTTGGAGAAATGAATG | 60°C |
| *RT-gyrB RV s.a.* | CAAATGTTTGGTCCGCTT |  |
| *RT-gy FW s.c* | CCTCCAGCATGTAATACC | 60°C |
| *RT-gyrB RV s.c* | GAGGCTTGCACCATTTAG |  |
| *RT-gyrB FW s.e* | TGACGAGGCATTAGCAGGTT | 60°C |
| *RT-gyrB RV s.e* | GTGAAGACCGCCAGATACTTT |  |

10 μM primer concentration in PCRs and 0.1 µM in RT PCR.

We used the following conditions PCR conditions: initial denaturation at 94°C for 30 seconds, followed by 30 cycles of denaturation at 94°C for 30 seconds, annealing temperatures (see Table) and extension at 68°C for 96 seconds. A final extension was carried out at 68°C for 5 minutes. The reactions were conducted on a Bio-Rad T100 Thermal Cycler (Bio-Rad Laboratories, USA) using a 96-well non-skirted PCR plate with oriented labelling (VWR, Germany).

**Supplementary Table 2.** Simplified synergy testing with 42 selected isolates for combination of cefazolin and gentamicin.

| **Species (Strain ID)** | **Gentamicin**  **MIC** | **Cefazolin MIC** | **Synergy-test with gentamicin 12 mg/L and variable cefazolin concentrations, see below** | | | | | | | |
| --- | --- | --- | --- | --- | --- | --- | --- | --- | --- | --- |
|  | **mg/L** | **mg/L** | **8 mg/L** | **4 mg/L** | **4 mg/L** | **2 mg/l** | **3 mg/L** | **1.5 mg/L** | **2 mg/L** | **1 mg/L** |
| *S. epidermidis (60)* | 128 | >256 | **+** | **+** |  |  |  |  |  |  |
| *S. capitis (78)* | 32 | >256 | **+** | **+** |  |  |  |  |  |  |
| *S. haemolyticus (83)* | 128 | >256 | **+** | **+** |  |  |  |  |  |  |
| *S. epidermidis (119)* | 128 | 96 | **+** | **+** |  |  |  |  |  |  |
| *S. capitis (134)* | 64 | 64 | **-** | **+** |  |  |  |  |  |  |
| *S. capitis (108)* | 32 | 48 | **+** | **+** |  |  |  |  |  |  |
| *S. capitis (99)* | 32 | 24 | **-** | **-** |  |  |  |  |  |  |
| *S. capitis (30)* | 32 | 12 | **-** | **-** |  |  |  |  |  |  |
| *S. epidermidis (39)* | 256 | 8 |  |  | **+** | **+** |  |  |  |  |
| *S. epidermidis (98)* | 256 | 8 |  |  | **+** | **+** |  |  |  |  |
| *S. capitis (107)* | 32 | 8 |  |  | **-** | **-** |  |  |  |  |
| *S. epidermidis (5)* | 256 | 8 |  |  | **+** | **+** |  |  |  |  |
| *S. epidermidis (36)* | 256 | 8 |  |  | **+** | **+** |  |  |  |  |
| *S. epidermidis (114)* | 256 | 8 |  |  | **+** | **+** |  |  |  |  |
| *S. capitis (10)* | 32 | 6 |  |  |  |  | **-** | **+** |  |  |
| *S. epidermidis (16)* | 256 | 6 |  |  |  |  | **+** | **+** |  |  |
| *S. epidermidis (63)* | 256 | 6 |  |  |  |  | **+** | **+** |  |  |
| *S. capitis (66)* | 32 | 6 |  |  |  |  | **-** | **+** |  |  |
| *S. epidermidis (71)* | 128 | 6 |  |  |  |  | **+** | **+** |  |  |
| *S. capitis (76)* | 32 | 6 |  |  |  |  | **+** | **+** |  |  |
| *S. capitis (85)* | 256 | 6 |  |  |  |  | **+** | **+** |  |  |
| *S. epidermidis (89)* | 256 | 6 |  |  |  |  | **+** | **+** |  |  |
| *S. capitis (127)* | 32 | 6 |  |  |  |  | **+** | **+** |  |  |
| *S. epidermidis (155)* | 512 | 6 |  |  |  |  | **+** | **+** |  |  |
| *S. epidermidis (158)* | 128 | 6 |  |  |  |  | **+** | **+** |  |  |
| *S. epidermidis (163)* | 512 | 6 |  |  |  |  | **+** | **+** |  |  |
| *S. epidermidis (165)* | 512 | 6 |  |  |  |  | **+** | **+** |  |  |
| *S. capitis (22)* | 32 | 6 |  |  |  |  | **-** | **-** |  |  |
| *S. capitis (129-b)* | 32 | 6 |  |  |  |  | **+** | **+** |  |  |
| *S. capitis (42)* | 32 | 4 |  |  |  |  |  |  | **-** | **+** |
| *S. epidermidis (51)* | 256 | 4 |  |  |  |  |  |  | **+** | **+** |
| *S. capitis (59)* | 32 | 4 |  |  |  |  |  |  | **+** | **+** |
| *S. epidermidis (112)* | 256 | 4 |  |  |  |  |  |  | **+** | **+** |
| *S. epidermidis (128)* | 64 | 4 |  |  |  |  |  |  | **+** | **+** |
| *S. epidermidis (141)* | 256 | 4 |  |  |  |  |  |  | **+** | **+** |
| *S. capitis (53)* | 128 | 4 |  |  |  |  |  |  | **+** | **+** |
| *S. epidermidis (56)* | 256 | 4 |  |  |  |  |  |  | **+** | **+** |
| *S. epidermidis (91)* | 128 | 4 |  |  |  |  |  |  | **+** | **+** |
| *S. epidermidis (100)* | 256 | 4 |  |  |  |  |  |  | **+** | **+** |
| *S. capitis (106)* | 32 | 4 |  |  |  |  |  |  | **+** | **+** |
| *S. epidermidis (110)* | 128 | 4 |  |  |  |  |  |  | **+** | **+** |
| *S. epidermidis (160)* | 64 | 4 |  |  |  |  |  |  | **+** | **+** |

Forty-two isolates were screened for synergy using a fixed gentamicin concentration of 12 mg/L and varying cefazolin concentrations ranging from 1 to 8 mg/L. The results, obtained from three biological replicates, are presented as binary values: growth (+) or no growth (-).

**Supplementary Table 3.** Induction experiments

| **Species (strain ID)** | **Gradient MIC test**  **MIC cefazolin (mg/L)** | **Microbroth dilution assay**   - **without antibiotics**   **MIC cefazolin (mg/L)** | **Microbroth dilution assay**   - **induction with cefazolin 1 mg/L**   **MIC cefazolin (mg/L)** |
| --- | --- | --- | --- |
| *E. coli ATCC 25922* |  | 2 (target value) |  |
| *S. capitis* (1) | 4 | 4/4/4 | 8/8/8 |
| *S. capitis* (42) | 4 | 4/4/4 | 4/4/4 |
| *S. capitis* (53) | 4 | 4/4/4 | 8/8/8 |
| *S. capitis* (59) | 4 | 4/4/4 | 4/4/4 |
| *S. capitis* (106) | 4 | 2/2/2 | 4/4/4 |
| *S. capitis* (4) | 4 | 4/4/4 | 4/4/4 |
| *S. capitis* (18) | 4 | 2/2/2 | 2/2/2 |
| *S. capitis* (6) | 4 | 2/2/2 | 4/4/4 |
| *S. capitis* (54) | 4 | 2/2/2 | 4/4/4 |
| *S. capitis* (69) | 4 | 2/2/2 | 4/4/4 |
| *S. capitis* (97) | 4 | 4/4/4 | 4/4/4 |
| *S. capitis* (136) | 4 | 4/4/4 | 4/4/4 |
| *S. capitis* (12) | 4 | 2/2/2 | 2/2/2 |
| *S. capitis* (50) | 4 | 2/2/2 | 4/4/4 |
| *S. capitis* (11) | 4 | 4/4/4 | 4/4/4 |
| *S. epidermidis* (51) | 4 | 8/8/8 | 8/8/8 |
| *S. epidermidis* (91) | 4 | 4/4/4 | 8/8/8 |
| *S. epidermidis* (112) | 4 | 4/4/4 | 8/8/>64 |
| *S. epidermidis* (128) | 4 | 8/8/16 | 8/8/16 |
| *S. epidermidis* (141) | 4 | 4/4/4 | 4/4/4 |
| *S. epidermidis* (100) | 4 | 4/4/8 | 8/8/8 |
| *S. epidermidis* (110) | 4 | 4/4/8 | 4/4/8 |
| *S. epidermidis* (160) | 4 | 4/4/4 | 4/4/4 |
| *S. epidermidis* (56) | 4 | **4/4/4** | **32/32/32** |
| *S. epidermidis* (116) | 4 | 4/4/8 | 4/4/4 |
| *S. epidermidis* (7) | 4 | 8/8/8 | 8/8/8 |
| *S. epidermidis* (48) | 4 | 8/8/8 | 8/8/8 |
| *S. epidermidis* (70) | 4 | 32/32/32 | 32/64/64 |
| *S. epidermidis* (72) | 4 | 4/4/4 | 8/8/8 |
| *S. epidermidis* (79) | 4 | 4/4/4 | 8/8/8 |

**Supplementary Table 4.** Fold changes in *mecA* gene expression relative to the housekeeping gene gyrase subunit B (*gyrB*) in relation to oxacillin and cefazolin MIC values in selected oxacillin-resistant CoNS and MRSA isolates

| **Species (Strain ID)** | **Oxacillin MIC (mg/L)** | **Cefazolin MIC**  **(mg/L)** | ***mecA* Expression Fold**  **(Mean ± SD)** | ***mecA* Ct Values**  **(Mean ± SD)** | ***gyrB* Ct Values**  **(Mean ± SD)** |
| --- | --- | --- | --- | --- | --- |
| S. capitis (30) | >256 | 16 | 1.33 ± 1.75 | 24.52 ± 3.92 | 23.19 ± 2.63 |
| *S. epidermidis (114)* | >256 | 8 | 6.80 ± 7.94 | 22.99 ± 0.73 | 23.93 ± 3.99 |
| S. epidermidis (119) | >256 | 128 | 5.27 ± 2.79 | 18.18 ± 0.93 | 20.43 ± 1.73 |
| *S. epidermidis (117)* | 64 | 8 | 1.02 ± 0.70 | 21.56 ± 0.57 | 21.34 ± 0.79 |
| *S. capitis (95)* | 32 | 16 | 4.64 ± 3.27 | 18.78 ± 1.71 | 20.08 ± 0.64 |
| *S. epidermidis (94)* | 16 | 4 | 0.30 ± 0.09 | 20.87 ± 0.36 | 19.09 ± 0.57 |
| *S. capitis (154)* | 4 | 4 | 0.28 ± 0.11 | 22.86 ± 1.77 | 20.94 ± 2.36 |
| *S. capitis (25)* | 4 | 4 | 1.50 ± 0.87 | 22.07 ± 0.20 | 22.46 ± 1.20 |
| *S. epidermidis (9)* | 2 | 1 | 0.85 ± 0.35 | 19.63 ± 0.47 | 19.33 ± 0.52 |
| *S. aureus (2087)* | 128 | 256 | 3.36 ± 2.20 | 17.44 ± 0.92 | 19.65 ± 0.72 |
| *S. aureus (2009)* | >256 | >256 | 2.19 ± 0.31 | 16.54 ± 0.92 | 17.66 ± 1.00 |
| *S. aureus (674)* | >256 | >256 | 3.04 ± 1.18 | 18.70 ± 1.25 | 19.55 ± 0.60 |
| *S. aureus (458)* | >256 | >256 | 1.38 ± 0.47 | 17.62 ± 0.31 | 18.37 ± 0.57 |

**References:**

1. Murakami K, Minamide W, Wada K, et al. Identification of methicillin-resistant strains of staphylococci by polymerase chain reaction. *Journal of clinical microbiology* 1991;29(10):2240–44.

2. Vakulenko SB, Donabedian SM, Voskresenskiy AM, et al. Multiplex PCR for detection of aminoglycoside resistance genes in enterococci. *Antimicrob Agents Chemother* 2003;47(4):1423–6. doi: 10.1128/aac.47.4.1423-1426.2003

3. Liu P, Xue H, Wu Z, et al. Effect of bla regulators on the susceptible phenotype and phenotypic conversion for oxacillin-susceptible mecA-positive staphylococcal isolates. *J Antimicrob Chemother* 2016;71(8):2105–12. doi: 10.1093/jac/dkw123 [published Online First: 20160506]
